# Label-free spectral imaging to study drug distribution and metabolism in single living cells

**DOI:** 10.1038/s41598-021-81817-0

**Published:** 2021-02-01

**Authors:** Qamar A. Alshammari, Rajasekharreddy Pala, Nir Katzir, Surya M. Nauli

**Affiliations:** 1 Department of Biomedical & Pharmaceutical Sciences, Harry and Diane Rinker Health Science Campus, Chapman University, Irvine, CA 92618 USA; 2 Department of Pharmacology and Toxicology, Faculty of Pharmacy, Northern Border University, Rafha, Kingdom of Saudi Arabia; 3 Department of Medicine, University of California Irvine, Irvine, CA 92868 USA; 4 Applied Spectral Imaging, Carlsbad, CA 92008 USA

**Keywords:** Spectrophotometry, Target validation

## Abstract

During drug development, evaluation of drug and its metabolite is an essential process to understand drug activity, stability, toxicity and distribution. Liquid chromatography (LC) coupled with mass spectrometry (MS) has become the standard analytical tool for screening and identifying drug metabolites. Unlike LC/MS approach requiring liquifying the biological samples, we showed that spectral imaging (or spectral microscopy) could provide high-resolution images of doxorubicin (dox) and its metabolite doxorubicinol (dox’ol) in single living cells. Using this new method, we performed measurements without destroying the biological samples. We calculated the rate constant of dox translocating from extracellular moiety into the cell and the metabolism rate of dox to dox’ol in living cells. The translocation rate of dox into a single cell for spectral microscopy and LC/MS approaches was similar (~ 1.5 pM min^−1^ cell^−1^). When compared to spectral microscopy, the metabolism rate of dox was underestimated for about every 500 cells using LC/MS. The microscopy approach further showed that dox and dox’ol translocated to the nucleus at different rates of 0.8 and 0.3 pM min^−1^, respectively. LC/MS is not a practical approach to determine drug translocation from cytosol to nucleus. Using various methods, we confirmed that when combined with a high-resolution imaging, spectral characteristics of a molecule could be used as a powerful approach to analyze drug metabolism. We propose that spectral microscopy is a new method to study drug localization, translocation, transformation and identification with a resolution at a single cell level, while LC/MS is more appropriate for drug screening at an organ or tissue level.

## Introduction

A standard imaging gives the intensity at each pixel of the image, and a conventional spectrometer provides spectral information of chemical substances. Spectral imaging merges these two elements by providing an intensity and spectral information for each pixel of an image^1^. Spectral information from images has been used to distinguish physiological changes and disease states from tissue samples from various organs^2^. However, spectral imaging is yet to be confirmed for use in drug development. While spectral imaging might provide a variety of image information as seen in Raman or fluorescence microscopy technique, we here focused on the use of the spectral microscopy that uses the absorption and emission spectral information.

The spectral imaging system was first introduced by NASA and the remote earth sensing community for monitoring temperature change and weather pattern. The spectral imaging has since been used for utilizing in academic and biomedical applications. Spectral imaging has been broadly developed as a useful quantitative technique in biomedical research. For example, it has been proposed for the in vivo melanoma detection based on color dispersal and morphology of the lesions^3^, determination of the blood–brain barrier opening process for drug release^4^, examination of autophagy and apoptosis regions^5^ and differential observation of vascular tissues from non-vascular regions of skin^6^. In addition, spectral imaging is also used to monitor the dynamic alterations of the tumor vasculature in living animals^7^ and visualize drugs absorption delivered through topical application^8^. Such approaches with unique spectral characteristics of molecules can provide detailed information with a high spatial and temporal resolution.

Drug metabolite identification can enhance our understanding of pharmacological response to improve lead compounds, distinguish new biochemical substances and reduce drug toxicity/interaction^9^. Quadrupole time-of-flight (QTOF) liquid chromatography and mass spectrometry (LC/MS) are commonly used to identify drug metabolites. These methods provide distinguishable quantitation between parental drug and its metabolites, based on their mass-to-charge ratio (m/z). The matrix-assisted laser desorption ionization time-of-flight mass spectrometry (MALDI-TOF/MS) is another analytical system of identification and characterization based on the fast and precise evaluation of the mass of molecules. However, the major drawback of these approaches is that they will destroy the biological samples. Besides, none of these techniques allows subcellular localization of the organic substances in the biological samples without the use of labeling^10^.

Doxorubicin (dox) is one of the anthracycline molecules. It is one of the most effective and prescribed anti-cancer drug. The anticancer action of dox depends mostly on its direct interaction with nucleic acids, leading to DNA damage and inhibition of DNA synthesis^11^. In general, dox is used in the medical treatment for breast cancer, leukemia, lymphomas, and sarcomas^12^. However, due to its potential metabolite, the use of dox has resulted in various side effects.

Evaluation of drug and its metabolite requires either labeling the drug molecule or liquifying the biological sample, despite the fact that such labeling is not stable and can alter the functionality of the molecules^1,13,14^. In our current study, we chose non-labelled doxorubicin (dox) as our experimental drug due to its known spectral characteristic at 592 nm^15^. We measured the spectrum at the range of 500 nm to 750 nm with a single triple band filter called “SKY” to detect the dox level in the samples. We also characterized dox metabolite, non-labelled doxorubicinol (dox’ol)^16^. Rhodamine 6G was also used to confirm our findings on dox, because rhodamine shares very similar emission spectra with dox. Our spectral microscopy data were confirmed and compared with the standard QTOF-LC/MS method. While both methods were corresponded to each other, we also found that the spectral microscopy had a better advantage to study cellular compartmentalization of small molecules.

## Results

### The spectral microscopy system indicates localization of doxorubicin (dox) and its metabolite doxorubicinol (dox’ol) to cell nucleus

**Figure 1:**
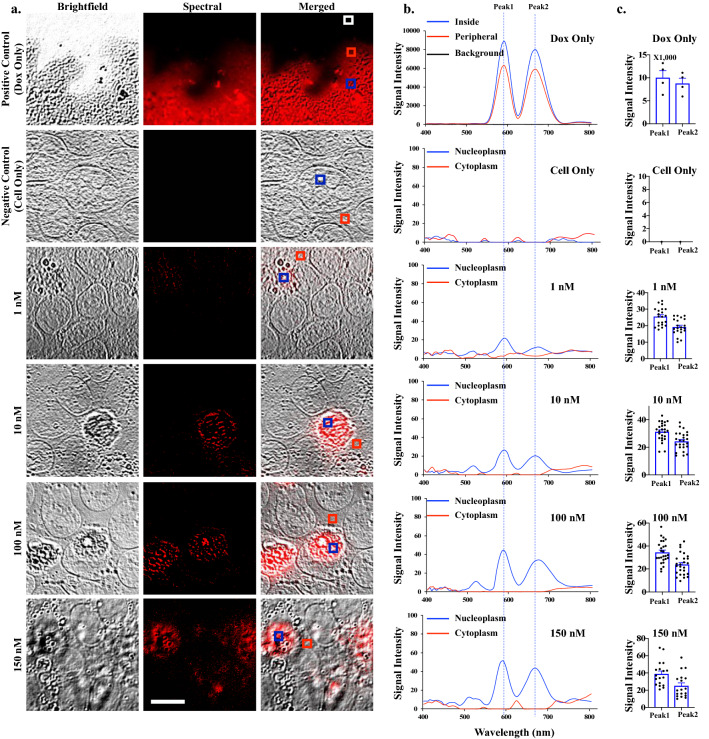
Spectral imaging on doxorubicin (dox). **(a)** Brightfield, emission spectral and merged images of dox-only (dissolved dox as a positive control), non-treated cells (negative control), and 18-h dox-treated cells at various concentrations. Cells were then fixed and imaged. White box = background; red box = lower signal intensity; blue box = higher signal intensity for dox. **(b)** The graphs show the spectra characteristics of dox, showing peaks at 592 nm (Peak1) and 670 nm (Peak2). **(c)** The bar graphs exhibit the variations of the intensity data points for Peak1, which was higher than Peak2. N = 4 (control groups) and N $\ge$ 20 (treatment groups). Scale Bar = 10 μm.

The pure dox spectral characteristics were captured using the SKY filter in cell-free (positive control) and fixed-cell systems. The spectral images revealed dox location in the cell, which mainly localized in the nucleus (Fig. 1a). The characteristics spectra of the pure dox were recorded to have peaks at 592 and 670 nm. The spectral characteristics of dox were then uploaded and stored in the spectral library, and they were recalled and pseudo-colored during experimental cell analysis. The intensity in each pixel of the spectral was compared among the background (negative control) and different concentrations of dox (Fig. 1b; see “Materials and methods”). Importantly, we were able to identify and quantify the amount and location of dox within single cells at a lower concentration. Please note that while we treated the cells with 1 nM of dox, this did not imply that 1 nM of dox was detected inside the cells. This was discussed below. The intensity variations of the dox wavelengths were quantified for each dox concentration (Fig. 1c). The spectra intensity was increased as higher concentrations of dox were used. Interestingly, we also observed a potential peak at 520 nm in the cells treated with a higher dose of dox. Because this peak was not observed in cell-free system, we hypothesized that 520 nm might represent a metabolite peak of dox.

**Figure 2:**
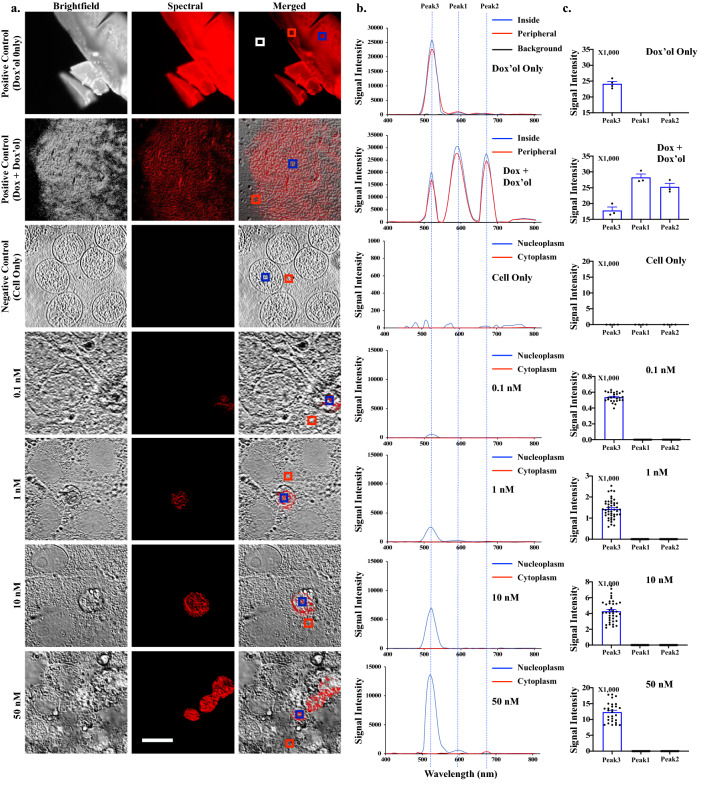
Spectral imaging on doxorubicinol (dox’ol). **(a)** Brightfield, emission spectral and merged images of dox’ol-only (dissolved dox’ol as a positive control), 2:3 of dox’ol:dox (positive control), non-treated cells (negative control), and 18-h dox’ol-treated cells at various concentrations. Cells were then fixed and imaged. White box = background; red box = lower signal intensity of dox’ol; blue box = higher signal intensity for dox’ol. **(b)** The graphs show the spectra characteristics of dox’ol, showing peak at 520 nm (Peak3). Dox is shown with peaks at 592 nm (Peak1) and 670 nm (Peak2). **(c)** The bar graphs exhibit the variations of the intensity data points for Peaks1, 2, and/or 3. N = 3 (control groups) and N $\ge$ 20 (treatment groups). Scale Bar = 10 μm.

Dox is known to be metabolized to doxorubicinol (dox’ol)^16–18^. To investigate if the 520 nm peak was dox’ol, we scanned the wavelengths of dox’ol only, different concentrations of dox’ol, and dox/dox’ol mixture (Fig. 2a). We could confirm that the spectral peak of the dox’ol was at 520 nm (Fig. 2b). The spectral characteristics of dox’ol were uploaded and stored in the spectral library, and they were recalled and pseudo-colored in the other separate images for further analyses. In addition, the background spectra with or without the cells were recorded to distinguish spectral intensity at 520 nm peak. The intensity variations of the dox’ol wavelengths were next quantified for each dox’ol concentration (Fig. 2c). Cells treated with different concentrations of dox’ol showed the location of dox’ol within the cell samples, which was concentrated mainly in the nucleoplasm area. Hence, we confirmed for the first time the ability of spectral microscopy system to track and identify a high image resolution of parental drug dox and its metabolite dox’ol in single cells.

### The spectral microscopy was validated with spectrophotometer and LC/MS

**Figure 3:**
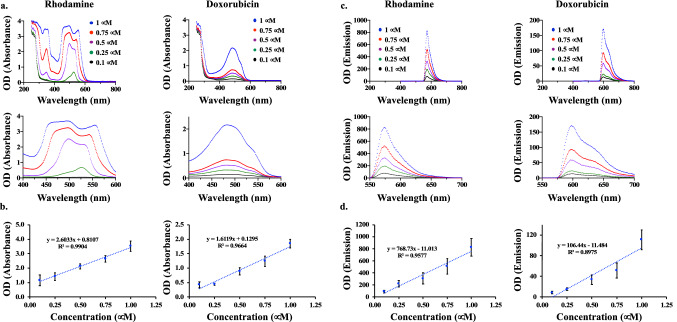
Absorbance and emission optical density of rhodamine and doxorubicin. **(a)** Spectrophotometer UV–VIS wavelengths of rhodamine 6G at a peak of 530 nm and doxorubicin at 483 nm. Each line presents a different concentration. The lower panels show the enlarged scale. **(b)** Linear regression analysis for rhodamine and dox. **(c)** Fluorescence wavelengths of rhodamine showing a peak at 580 nm and dox at 592 nm. The lower panels show the enlarged scale. **(d)** Linear regression analysis for the respective peaks of rhodamine and dox. N = 3.

To independently verify the spectra microscopy studies of dox, we re-examined the dox spectra using spectrophotometry, in addition to QTOF and HPLC for quantitation purposes. Rhodamine 6G was also used to validate our findings on dox, because rhodamine shares similar spectra with dox and importantly is readily fluorescent for imaging purposes. Specifically, rhodamine and dox had very similar absorption and emission spectra characteristics. We looked at the scans of absorption spectra for rhodamine and dox (Fig. 3a). Although we observed the linearity of the absorption at 483 nm for dox (Fig. 3b), this had no significant property in the spectral microscopy. Spectrophotometry scans further indicated that optical densities of emission for dox and rhodamine had peaks at the corresponding spectra of 592 and 580 nm (Fig. 3c). We next confirmed the linearity of the intensity and the concentration suggesting that the peak of dox at 592 nm could be used for quantitation purpose in the spectral microscopy method (Fig. 3d). The wavelength at 592 nm in the spectral microscopy could very well represent emission spectra.

**Figure 4:**
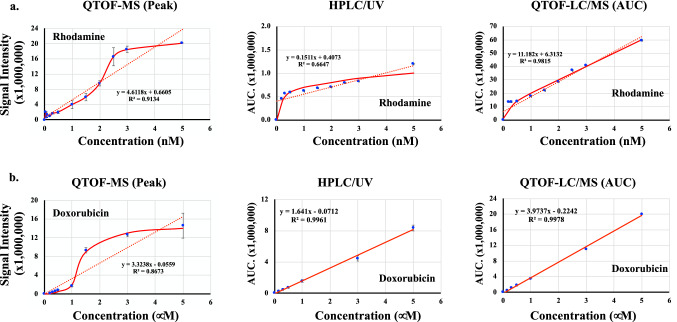
Mass spectroscopy and chromatography of rhodamine and doxorubicin. **(a)** Linear regression and curve-fit analyses of rhodamine were performed from QTOF-MS, HPLC–UV and QTOF-LC/MS data. The linearity was found at the precursor ion m/z 443 Da. The line of QTOF-MS reaches a plateau within 2.5 to 5 nM, which might be considered a sign of saturation. **(b)** Linear regression and curve-fit analyses of dox were done from QTOF-MS, HPLC–UV and QTOF-LC/MS data. The linearity observed at the precursor ion m/z 544 Da. The line of QTOF-MS reaches a plateau within 1.5 to 5 μM, which might reflect signal saturation. N = 3.

Having rhodamine as a control (Fig. 4a), we also evaluated dox using LC-alone, MS-alone and LC/MS in combination (Fig. 4b). Consistent with previous studies^19,20^, the linearity of the precursor ions for dox and rhodamine were found at m/z 544 Da and 443 Da, respectively. We later used this LC/MS standard curves to extrapolate the quantitative capability using the spectral microscopy.

### The spectral microscopy indicated that unlike dox and dox’ol, rhodamine was mainly localized in cytoplasm

**Figure 5:**
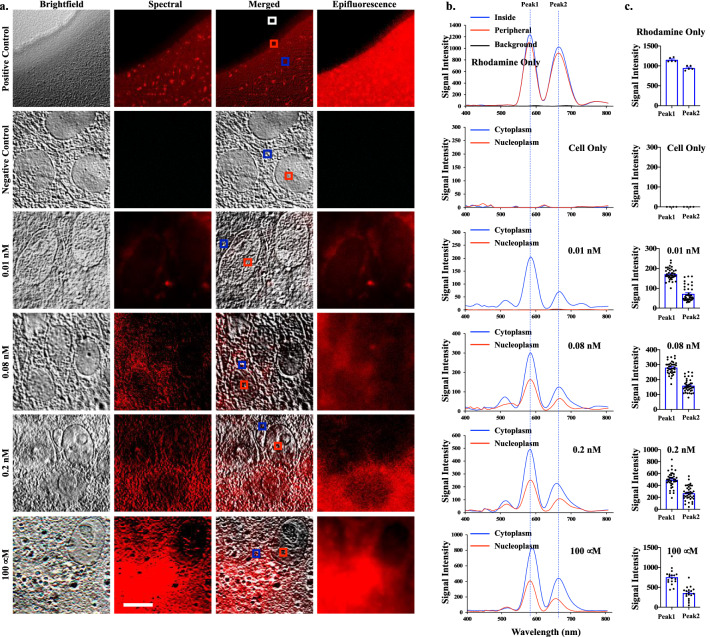
Spectral imaging on rhodamine. **(a)** Brightfield, emission spectral and merged images of rhodamine-only (dissolved rhodamine as a positive control), non-treated cells (negative control), and 18-h rhodamine-treated cells at various concentrations. Cells were then fixed and imaged. White box = background; red box = lower signal intensity of rhodamine; blue box = higher signal intensity for rhodamine. **(b)** The graphs show the spectra characteristics of rhodamine, showing peaks at 580 nm (Peak1) and 679 nm (Peak2). **(c)** The bar graphs exhibit the variations of the intensity data points for Peak1 and Peak2. N = 5 (control groups) and N $\ge$ 20 (treatment groups). Scale Bar = 10 μm.

We further validated the spectral microscopy system using rhodamine (Fig. 5a). While other chemicals with well-characterized spectra could also be used, the main reason rhodamine was selected to validate our system was that dox and rhodamine possessed similar spectra. Yet, the spectra (or wavelengths) of dox and rhodamine were distinct from each other. Dox has a peak spectrum at 592 nm, while rhodamine has a peak at 580 nm. Thus, the significant of our studies using spectral information (rather than fluorescent) was to eventually distinguish the identity of two closely resemble molecules; ie. dox vs. dox’ol.

The scanned spectral characteristics of rhodamine were uploaded and stored in the spectral library; they were recalled during a separate image analysis. In the spectral microscopy, the characteristics spectra of rhodamine were recorded with peaks at 580 and 679 nm (Fig. 5b), consistent with the previous studies^21^. The 580 nm might represent the emission spectra of rhodamine (Fig. 3). Unlike dox and dox’ol, however, rhodamine at lower concentrations tended to accumulate in the cytoplasm than in the nucleoplasm. The intensity peaks wavelengths were also quantified at different concentrations of rhodamine (Fig. 5c). Cells treated with higher concentrations of rhodamine showed an increase intensity, primarily on the cytoplasm and nucleoplasm to some extent. This was also confirmed with a regular fluorescence microscope. While we could confirm the spectral microscopy system identifying rhodamine, a regular fluorescence microscope might be easier to detect rhodamine which is readily fluorescent.

### The spectral microscopy had a linearity for quantification purposes

**Figure 6:**
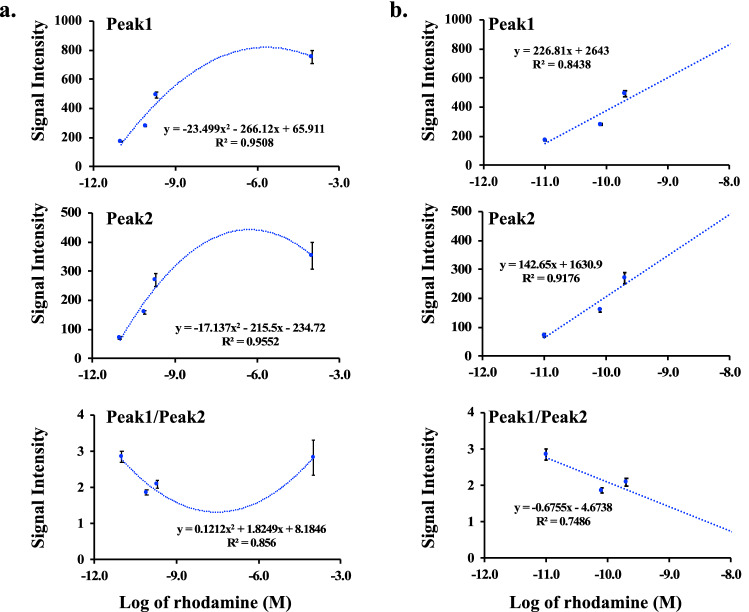
Analysis of linearity of rhodamine in spectral imaging. Linear regression data analyses of the cells treated with different concentrations of rhodamine 6G with spectral imaging. The graphs represent the average intensities of Peak1 at 580 nM, Peak2 at 679 nm, or the ratio of Peak1/Peak2, which included the saturation point at 100 μM **(a)** or without the saturation point **(b)**. N $\ge$ 20.

**Figure 7:**
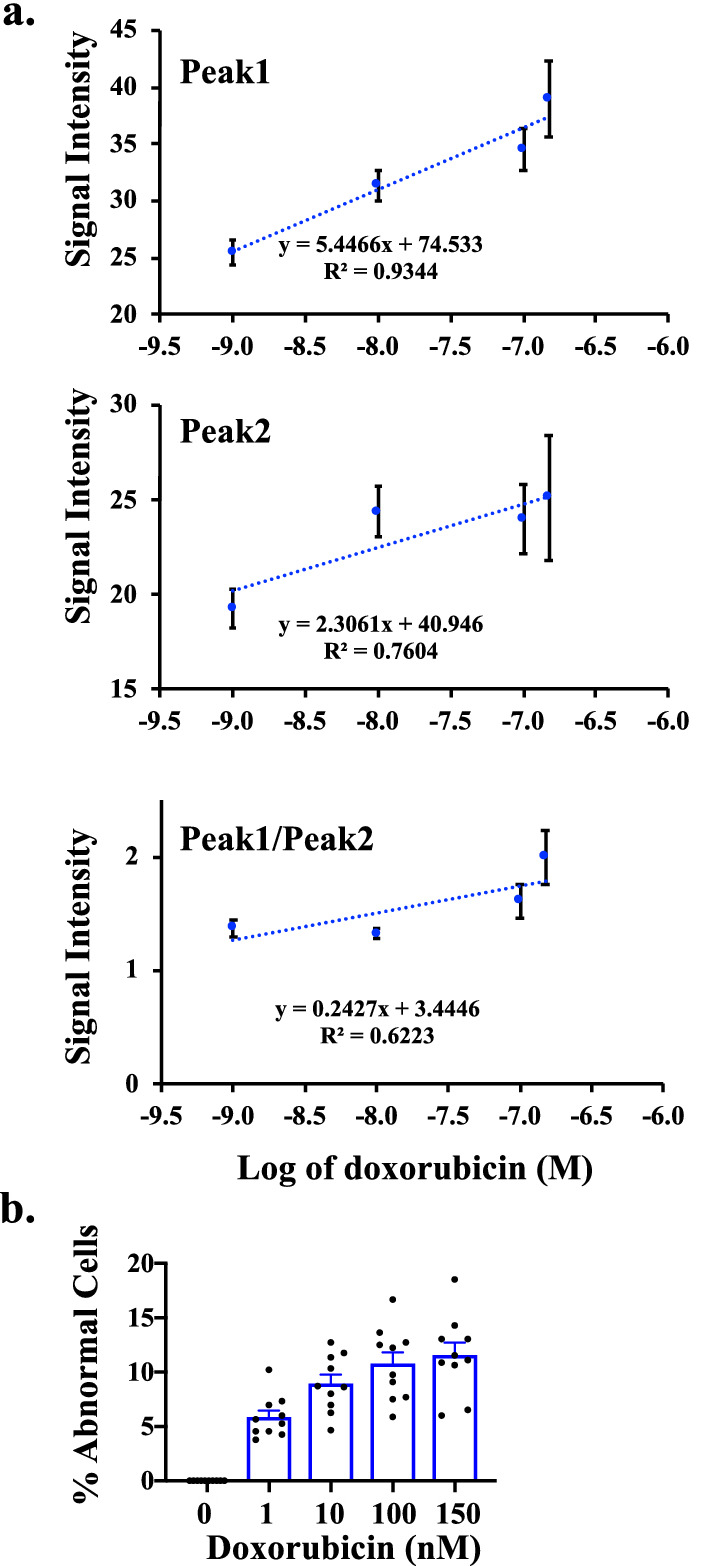
Analysis of linearity of doxorubicin in spectral imaging. **(a)** Linear regression for the cells treated with dox captured from spectral imaging. The graphs illustrate the average intensities of Peak1 at 592 nm, Peak2 at 670, and the ratio of Peak1/Peak2. N $\ge$ 20. **(b)** The bar graph displays the percentage of the abnormal cells (or dead cells) at different concentrations of dox. At least 50 cells were counted in N $\ge$ 9 independent experiments.

**Figure 8:**
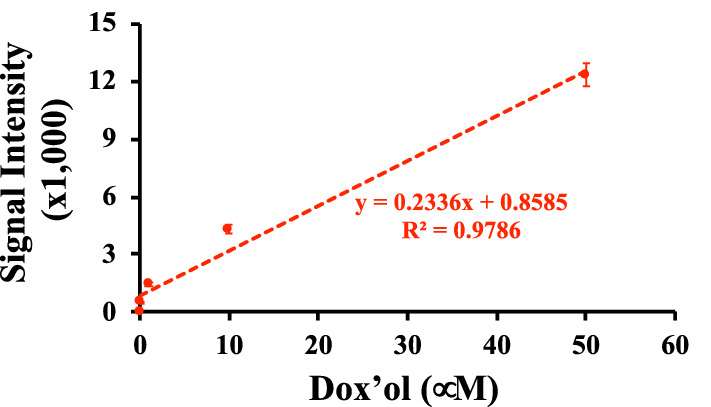
Analysis of linearity of doxorubicinol in spectral imaging. Linear regression analysis for the cells treated with dox’ol by spectral imaging. The graph illustrates the average intensities of the peak (520 nm). N = 3.

We evaluated if the spectral microscopy system could have the linearity to quantify rhodamine (Fig. 6), dox (Fig. 7) and dox’ol (Fig. 8). Our approach included analyses of each peak individually (Peak 1 and Peak 2) and potential interaction between peaks (Peak1/Peak2). For rhodamine, we analyzed the linearity with (Fig. 6a) and without (Fig. 6b) the oversaturated point. Note that the saturated point in our spectral microscopy system was used to confirm rhodamine using the standard fluorescence microscope (image not shown). The first major peak of rhodamine at 580 nm represented the linearity of spectral intensity that we could use for quantitation purposes for rhodamine. On the other hand, the linearity for dox was confirmed to be the major peak of 592 nm (Fig. 7a). Because dox is associated to induce cell apoptosis^22^, we took this opportunity to quantify cells with abnormal nucleus and/or smaller cell morphology (Fig. 7b). Our data confirmed that we were using dox compound having activity at a very low concentration of nM. Because dox’ol showed only one peak at 520, we proceeded to analyze this peak (Fig. 8). The linearity of spectral intensity of dox’ol peaks from the spectral microscopy was confirmed at 520 nm. Our analyses indicated that the spectral microscopy system had potential capability for quantification.

### The spectral microscopy could quantify the kinetic changes of intracellular levels of dox and dox’ol

To understand pharmacokinetics of dox, we first performed a time-lapse study (Supp Fig. S1) and analyzed the changes in spectral intensity for 0.01 nM of rhodamine (Supp Figs. S2, S3). Again, rhodamine was used as a control, because we could confirm its fluorescence easily using regular fluorescence microscope. There were increases in the amount of rhodamine with time in the cytoplasm area as depicted in the intensity increase. In the spectral microscopy system, the increase in the wavelength intensity included the peak at 515 nm, which was suspected to be rhodamine metabolite.

**Figure 9:**
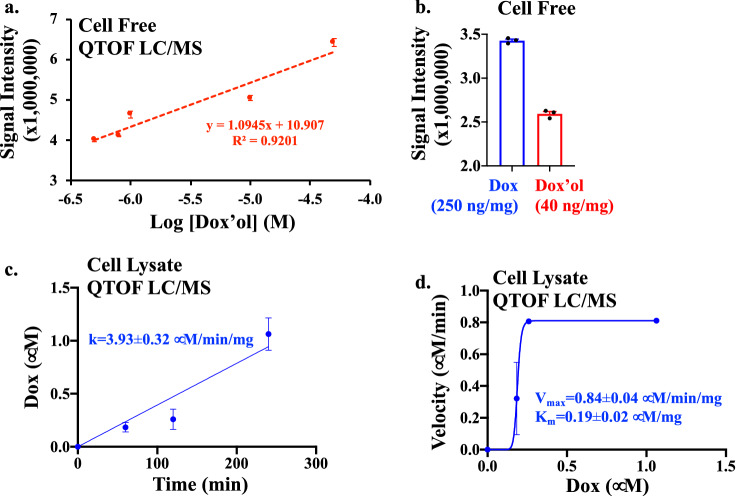
Pharmacokinetics of doxorubicin with a traditional method. **(a)** Linear regression analysis of dox’ol-only in the cell-free system was performed from QTOF-LC/MS data. The linearity of dox’ol was detected at the precursor ion m/z 546 Da. **(b)** The bar graph shows a pure mixture of dox and a dox’ol in cell-free system by Q-TOF LC/MS. **(c)** Cells treated with 2 μM dox were collected at different time points and analyzed for dox (m/z 544 Da). Dox influx into the cells was calculated. **(d)** Cells treated with 2 μM dox were collected at different time points and analyzed for dox’ol (m/z 544 Da). Enzyme kinetics (Vmax and Km) were calculated based on the linear influx of dox. N = 3.

For quantitation purposes, we used our prior studies using the traditional LC/MS method for dox (Fig. 4). For dox’ol, the linearity was found with the precursor ion at m/z 546 Da (Fig. 9a), consistent with a previous study^16^. We could differentiate two precursor ions (peaks) at m/z 544 Da (dox) and 546 Da (dox’ol) in cell-free system as another verification of our LC/MS (Fig. 9b) and cell lysate as a correlation for our pharmacokinetic studies for dox (Fig. 9c,d). The intracellular distribution of dox was calculated from LC/MS to be 3.93 ± 0.32 μM/min/mg of total protein with the Km of enzyme kinetics of 0.19 ± 0.02 μM/mg of protein.

**Figure 10:**
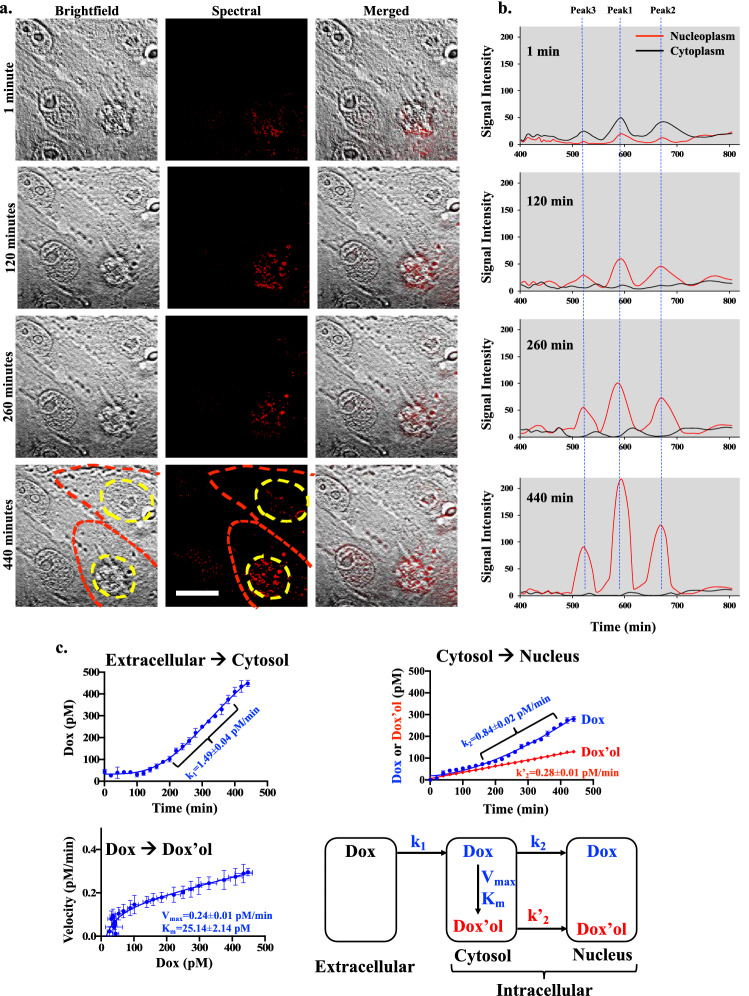
Metabolism of doxorubicin (dox) to doxorubicinol (dox’ol) in single living cells. **(a)** The time-lapse of brightfield, spectral and merged images are shown for cells treated with 1 nM dox for 440 min with red-line (cell border) and yellow-line (nucleus border). **(b)** The graphs depict the spectra intensity of peaks at 592 nm (Peak1; dox), 670 nm (Peak2) and 520 nm (Peak3; dox’ol). **(c)** The rate constants of dox influx into a cell (k_1_) and nucleus (k_2_) were calculated. The rate constant of dox’ol influx into a nucleus (k_2_′) was greater than k_2_. The enzyme kinetics of dox metabolism was calculated for their maximum velocity (Vmax) and Michaelis constant of dox affinity (Km). A 3-compartment model for dox metabolism is illustrated. N = 4. Scale Bar = 10 μm.

The time-lapse of dox was next performed in single cells for about 7 h (Supp Fig. S4). Quantitative analysis of the spectral intensity of 1 nM dox indicated that dox was first accumulated in the cytoplasm in the first hour (Supp Figs. S5, S6). Subsequently, a higher accumulation of dox was detected in the nucleoplasm (Fig. 10a). Of interest was the metabolite peak at 520 nm, which slowly and steadily increased (Fig. 10b). Given that dox (Fig. 1) and dox’ol (Fig. 2) were represented by the respective spectra peaks of 592 and 520 nm, we calculated the rate constants of dox and dox’ol distributions in the cells and kinetic conversion of dox to dox’ol (Fig. 10c). The rate of dox accumulation in the cytoplasm was 1.49 ± 0.04 pM min^−1^. In the cytosol, dox was converted to dox’ol with enzymatic characteristics of Vmax at 0.24 ± 0.01 pM min^−1^ and Km at 25.14 ± 2.14 pM. Both dox and dox’ol were then distributed to the nucleus with rate constants of 0.84 ± 0.02 pM min^−1^ and 0.28 ± 0.01 pM min^−1^, respectively.

## Discussion

Because evaluation of drug and its metabolite is an essential process to understand drug activity, stability, toxicity and distribution during drug development^23^, we compared the use of our novel technique to LC/MS, which is the standard analytical tool for screening and identifying drug metabolites^13^. Without labeling the drug and destroying the samples, we here demonstrated that spectral microscopy could allow us to analyze rates of drug metabolism and subcellular distribution. The significance of this method in drug discoveries is multiple. First, dox’ol is believed to contribute significantly to cardiotoxicity in cancer patients^16^. Because dox is still used as chemotherapy regimens, further investigation of dox metabolism is warranted. Second, the cardiotoxicity of dox’ol is more complicated than once thought. It is generally thought that dox’ol interferes with cardiac contraction primarily by inhibiting membrane-associated ion channel^17^. Our data showed that the long-term effect of dox’ol in the cell nucleus, which was previously unknown, could not be ignored.

Using a standard LC/MS approach, the intracellular distribution of dox was calculated to be 3.93 ± 0.32 Μm min^−1^ mg^−1^ of total protein (Fig. 9). To put this value into perspective from the spectral microscopy approach, we would need to assume that one mammalian cell has about 250 pg proteins^24^. This assumption would result in a rate constant of ~ 1.5 pM min^−1^·1.5 cell^−1^, compared to the microscopy approach of ~ 1.5 pM min^−1^ cell^−1^ (Fig. 10). Using QTOF-LC/MS, we calculated the Km of enzyme kinetics was 0.19 ± 0.02 μM mg^−1^ of protein (Fig. 9). With the same assumption of 250 pg protein/cell, the Km was converted to about ~ 25 pM per 100 cells by the traditional approach compared to spectral microscopy of ~ 25 pM per one cell (Fig. 10).

Comparing the discrepancy ratios between 1:1.5 cells for distribution rate constant and 1:500 cells for enzyme kinetic Km, we postulated that most of dox’ol metabolite were tightly bound to the DNA in the nucleus. As a result, the metabolism rate of dox to dox’ol might have been underestimated by 1 to 500 cells due to subcellular localization of dox’ol in the nucleus. During isolation from the cell lysate, the incomplete separation of nuclear dox/dox’ol could contribute to underestimation for the dox’ol formation by ~ 500 cells. It is important to note that the discrepancy did not imply that spectral microscopy provided more accuracies to calculate the respective distribution rate and enzyme kinetics. The reason was that both LC/MS and microscopy approaches were not equally comparable to one another. The spectral microscopy might provide a better resolution of drug disposition and metabolism at a single cell-resolution. On the other hand, LC/MS would provide a more robust analysis at an organ system level.

The spectral microscopy uses a combination of high-resolution imaging and spectroscopy techniques. This is a valuable approach that is currently under-utilized to study valuable biological and clinical samples. The advantages of the spectral microscopy include the following. *First*, it is not labor-intensive and time consuming for sample preparation to perform quantitative bioanalysis, compared to other methods. *Second,* there is no concern about matrix interfering with the drugs. Using a standard HPLC approach, matrix proteins, lipids or salts need to be removed either to prevent the column from clogging or to improve HPLC reliability, sensitivity and selectivity in the analysis. *Third*, the spectral microscopy approach requires no additional treatment to samples. In traditional fluorescence imaging, samples are to be tagged, treated or radio-labelled to increase the reliability of sample detection. The detection of drug and its metabolite becomes unlikely. *Fourth*, the high-resolution imaging provides spatial and temporal information of a drug and its metabolite. In contrast, a combination with spectroscopy gives valuable information of the drug metabolism for each pixel of the image. The disadvantage of the spectral microscopy is that unlike regular imaging which is relatively more straightforward, it involves changing the optical pass differences to collect the essential information for the entire image. In addition, the spectral microscopy for the use of quantitation will still depend on LC/MS to help calibrate the linearity of the spectral signal.

## Conclusion

Without labeling drugs and destroying the samples, spectral imaging is a novel method that allows us to analyze the dynamics of drug distribution and metabolism in single living cells (Supp Fig. S7). The spectral microscopy provides a high image resolution to track and identify doxorubicin and its metabolite doxorubicinol. The microscopy approach confirms that both doxorubicine and doxorubicinol are translocated to the nucleus at different rates, while rhodamine remains in cytoplasm. The use of novel spectral microscopy approach on doxorubicine, doxorubicinol and rhodamine is for the first time validated with a traditional approach of LC/MS.

## Materials and methods

### Materials

Anhydrous methanol HPLC grade (≥ 99.8%, lot# SHBG6650V), acetonitrile HPLC grade (≥ 99.9) and rhodamine 6G (MW: 479.01 g/mol, lot# BCBP8335V) were purchased from Sigma Aldrich (St. Louis, MO); doxorubicin (dox) hydrochloride (MW: 579.98 g/mol, lot# Q5L8K -FB) from TCI America; doxorubicinol (dox’ol) (hydrochloride; MW: 582 g/mol, lot# 22386) from Cayman Chemical Company; trifluoroacetic Acid (TFA) from EMD Millipore Corporation; formic acid (lot# 2595C389) and sucrose from Fisher Scientific (Fair Lawn, NJ); LL-CPK1 (ATCC; CL101.1TM) porcine renal epithelial cells from proximal tubule were obtained from American Type Culture Collection (ATCC; Manassas, VA). Trypsin, penicillin–streptomycin solution (lot# 04619001), phosphate buffered saline (PBS) (lot# 05319001), and Dulbecco's Modified Eagle Medium (DMEM) (lot# 20818006) were purchased from Corning (Manassas, VA). Fetal bovine serum (FBS) was obtained from Seradigm (Logan, UT); paraformaldehyde (PFA) from Electron Microscopy Services (Hatfield, PA); and, Mounting Media HistoChoice from Amresco. Lysis buffer from Thermo scientific (Rockford, IL) and protease inhibitor cocktail from Complete (Mannheim, Germany).

### Cell culture

LL-CPK1 cells were cultured to a confluent monolayer in DMEM supplemented with 10% FBS and 1% penicillin–streptomycin at 37 °C in 5% CO_2_ and 95% humidity. Cells were trypsinized (using a 0.05% solution of trypsin) regularly for passage when reached 70–90% confluence. For our experiments, cells were cultured to reach confluent before treated with the chemicals (dox, dox’ol, or rhodamine).

### UV–VIS and fluorescence spectrum detection

Spectrophotometer (SpectraMax M5 Microplate Reader) was used to read the ultraviolet–visible (UV–VIS) and fluorescence spectra for dox and rhodamine 6G. All molecules were prepared at different concentrations in methanol 0.1, 0.25, 0.5, 0.75, 1 μM. The wavelength range of 200–800 nm was used to read the UV spectrum for all the chemicals. The fluorescence spectrum range was 400–800 nm for dox and rhodamine.

### Detection and quantification of mass spectrometry and UV–VIS HPLC

Quadrupole Time-of-Flight (QTOF) Liquid chromatography-mass spectrometry (LC–MS) system (Bruker Impact II and Ultimate 3000 UPLC (Dionex)) was used to detect and quantify the higher and lower detection limits based on the mass spectra for both dox and rhodamine 6G. Mass spectrometry was performed with direct injection of different concentrations of dox and rhodamine at 150, 300, 450, 500 nM, 1, 1.5, 3, 5 μM and 0.2, 0.3, 0.5, 1.0, 1.5, 2.0, 2.5, 3.0, 5.0 nM, respectively. For the QTOF-LC/MS method, dox is prepared at the same concentrations and eluted using a gradient of solvent (A) 0.1% formic acid in Milli-Q water and solvent (B) 0.1% formic acid in acetonitrile. The flow rate was 0.3 mL/min, and the injection volume was 50 μL with a total acquisition time of 10 min (Table 1).

**Table 1:** RP-HPLC gradient for the separation of doxorubicin and doxorubicinol.

Time (min)	% A	% B	Flow rate (mL/min)
0	95	5	0.3
0	95	5	0.3
1.5	95	5	0.3
6	5	95	0.3
7	5	95	0.3
7.1	95	5	0.3
9	95	5	0.3

The reversed phase-high liquid chromatography (RP-HPLC) C18 column (Shimadzu 50 × 4.6 mm, 3 mm particle size) was used in the analysis. In addition, rhodamine was also prepared at the same concentrations and analyzed by using the isocratic elution method in 100% methanol and an injection volume of 50 μL. However, to quantify the limits of dox and rhodamine in the UV–VIS wavelength, the analytical Hitachi HPLC system was used. The gradient elution was conducted using mobile phase (A) 0.1% trifluoroacetic acid in Milli-Q water and (B) 0.1% trifluoroacetic acid in acetonitrile, with the same gradients of QTOF method for dox. Rhodamine was analyzed with the isocratic elution method that was used in QTOF. To validate that extra peak in dox (520 nm) representing the metabolite species of dox (dox’ol), different concentrations of the pure dox’ol (0.5, 0.8, 1, 10 and 50 μM) and a mixture of pure dox and dox’ol (80 and 20%, respectively) prepared for confirmation with the mass spectra by QTOF.

### Cell treatment

Cells were seeded into sterilized 22 × 22 mm coverslip (Globe Scientific), in 6 wells plate (Greiner bio-one Cellstar®, the total volume of 2 mL at each well) under normal growth conditions until reached 70–80% confluency. The cells were then treated with 100 μL of the selected chemical at different concentrations ranging from 1 to 150 nM for dox, 0.01 nM to 100 μM for rhodamine 6G, and 0.1 to 50 μM for dox’ol. The cells treated for a total incubation time of 18 h. Then, the cells washed three times by PBS, fixed the cells for 10 min in fixing solution (2.5 mL PFA, 7.5 mL PBS and 0.2 g sucrose) at room temperature. Afterward, the coverslip was placed on the slide that contained 25 μL of mounting media overnight at room temperature.

### Spectral imaging (spectral microscopy)

Spectral imaging instrument (ASI Spectral Imaging System) and Olympus microscope (Model BX61) were used. Manual image acquisition conducted with 60X magnification fields. Xenon arc lamp was used as our light source. Spectral filter (SKY) used to identify the wavelengths of the pure dox, dox’ol and rhodamine 6G. SKY filter produced a spectrum that had a background-subtracted spectral type. Each chemical was measured for its spectral characteristic, also known as spectral signature. After the spectrum of the background was subtracted, it was saved in a spectral library. The library was later on used for various spectral analysis tasks. For instance, to compare the dox spectrum with the background spectrum, we defined markers in the area that contained the chemical substance (dox) and in the area that did not have dox (background). Spectrum was collected from 400 to 800 nm with a step size of 20 nm. We subsequently displayed the two spectra (dox and background). The dox spectra were acquired by subtracting the background spectra. After that, we uploaded these spectra as libraries for further cell analysis. Subsequently, images of the cells that were treated with the chemicals were captured randomly by using SKY and brightfield filters. At least 10 field of views were captured per each concentration sample that was prepared. Afterward, we analyzed the images by using the spectral libraries of the chemicals and scanned all the areas to obtain the location that only matched within the chemical library. For dox, rhodamine, and dox’ol samples, we used SUN analysis (within the SpectraView software, Applied Spectral Imaging), performed Spectral UNmixing, divided a set of images into layers that matched to the original spectra (libraries). This allowed us to quantify the amount and identify the location of the material according to its original spectra.

**Figure 11:**
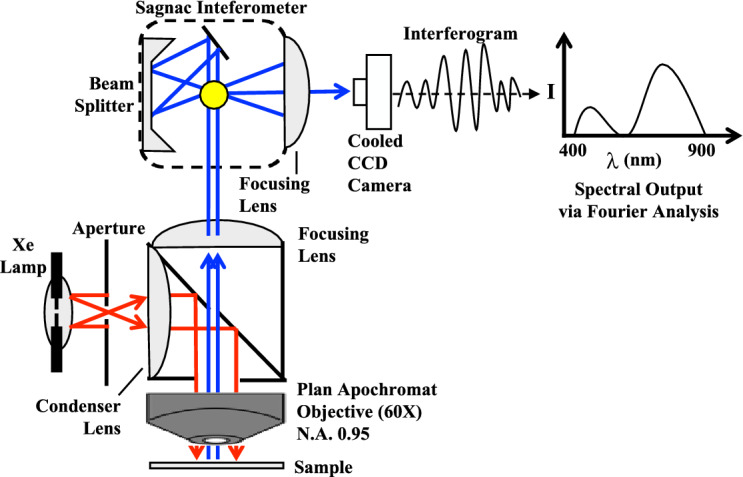
Diagram of the spectral microscopy set-up.

Our system used a standard microscopy set-up (Fig. 11). This set-up was widely available in most laboratories. The use of sagnac interferometer was also very common. The beam splitter split the light originating from the selected area in the sample into two beams. A set of mirrors led the beams down two paths of various lengths. At the end of the paths, the two beams combined. At the point when the two beams merged, they were superimposed. The total intensity of these two superimposed beams was a function of the variance in the distance between the two paths. This path variance was called the Optical Path Difference (OPD). The intensity of the merged beam was then captured by the Charged-Coupled Device (CCD) camera. Each measurement was called a frame, which was a simple gray level image measured by the CCD camera. Many frames were acquired, using different OPDs in each pixel to build a spectral image. At each OPD, this process arose simultaneously for each pixel in the image. At each pixel, all the frames acquired for that pixel were used to build an interferogram. An interferogram was an illustration of the light intensity, which might vary with each altering OPD. Each pixel’s interferogram was transformed into that pixel’s spectrum. The interferograms of all the pixels taken together permitted the rebuilding of the entire image’s spectrum. Fourier transformation allowed us to convert the interferogram from each pixel into a pixel’s spectrum. Through merging Fourier transformation with spectroscopy, we were able to further analyze the spectra of microscopic samples.

### Time-lapse imaging

LLCPK cells were grown in six-well plate and treated with 1 nM for dox or 0.01 nM rhodamine. Cells were captured directly (without fixation) using brightfield and spectral imaging every 20 min for 8 h.

### Cell Lysate

To validate that extra peak in dox (520 nm) representing the metabolite species of dox (dox’ol), different concentrations of the pure dox’ol (0.5, 0.8, 1, 10 and 50 μM) and a mixture of pure dox and dox’ol (80 and 20%, respectively) prepared to check the mass spectra by QTOF. In a different experiment, cells were treated with 2 μM of dox and dox’ol separately at different incubation times (18, 26, 42 h). Then, cells were washed using PBS by keeping the plates on the ice for all the steps. After that, 200 mL of lysis buffer supplemented with protease inhibitor cocktail were added to the cells; the cells were isolated by using a cell scraper (VWR; Radnor, PA) and transferred the lysate to a 5 mL Eppendorf. Afterward, the cells were centrifuged at 14,000×*g* for 10 min at 4 °C. Finally, the supernatant was collected by avoiding the pellet and put into new vials for QTOF analysis.

### Metabolite identification

**Figure 12:**
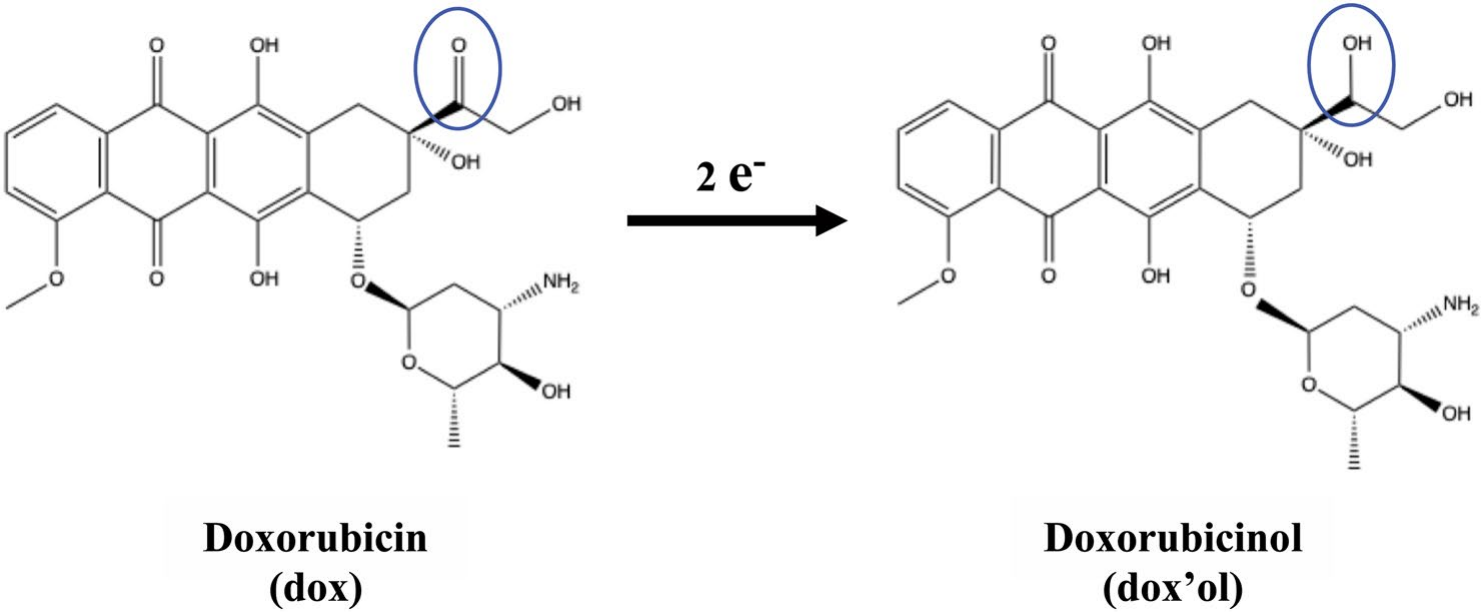
Diagram of dox converted to dox’ol at the side chain of the C-13 carbonyl group.

The major metabolite of dox is dox’ol, and alcohol metabolite is the primary metabolic route of dox metabolism to dox'ol^16–18^. Two-electrons of a side chain of the C-13 carbonyl group (Fig. 12) were reduced by aldo–keto reductase (AKR) and short-chain dehydrogenase/reductase^25,26^. To confirm the peak at 520 nm was belonged to dox’ol, cells were treated with 100 μL dox’ol at different concentrations (0.1, 1, 10, 50 μM) with a total volume of 2 mL for 18 h. Then, cells were washed three times with PBS and fixed for 10 min in fixing solution at room temperature. After that, cells were mounted with 25 μL of Mounting Media overnight at room temperature. Finally, spectral imaging was used for further analysis by using the same approach of dox (manual image acquisition with 60X magnification fields, use SKY and bright-field filters, at least 10 capturing for each concentration).

### Subcellular drug distribution

After capturing the brightfield image and spectral information of the cells, we analyzed the image using the spectral (or wavelength) library. We made boundaries for the nucleus as our region of interest (ROI) from the brightfield image (Supp Fig. S7). Because the cells were fully confluent, anything outside the ROI was considered cytoplasm. Please note that the spectral information initially contained no image; it only contained absorption/emission information, similar to a standard spectrophotometer. However, the spectral information containing dox-specific spectral could be extracted and converted to an image. The positive control provided information of dox-specific spectral, which had been stored in the library (after background subtraction). We could recall the spectral that resembled the spectra library (i.e. dox-specific spectra). These spectra were projected as a pseudocolored image. Thus, each pixel of the image contained its own spectra information; in this case, the information was the dox-specific spectra. The pseudocolored image obtained and processed from the spectra information was then transferred to the brightfield image (containing ROI for nuclei). The superimposed pseudocolored and brightfield image was saved and labeled as a “merged” image. Thus, in the merged image we could obtain both ROI and spectral identity of dox to distinguish dox localization in nucleoplasm or cytoplasm.

### Image and statistical analyses

Most of our image and statistical analyses were conducted by using Spectral Imaging software (GenASIs) version 7.2.7.34276 and GraphPad Prism software version 8. Microsoft Excel software version 16 was also used for linear regression analysis to obtain a standard calibration curve and linear equation. Comparisons between two groups were analyzed with two-tailed Student t-tests. Drawing of computer setup (Fig. 11) was made using Microsoft PowerPoint software version 16. ChemDraw version 18 was used to draw the chemical structures (Fig. 12). The spectral microscopy workflow (Supp Fig. S7) was self-created from the paid-online subscription at BioRender (Invoice #CEE5813A-0003; Receipt #2338-5420).

The intracellular distribution of dox was calculated from data collected at different time points. Unlike LC/MS approach where different samples were used at each time point, spectral imaging allowed us to use a single same sample for multiple time points. We obtained the rate constant (k_1_) of dox influx into a cell using spectral imaging approach (or cell lysate for LC/MS approach). Because one molecule of dox is converted to one molecule of dox’ol, the k_1_ was calculated from the slope of total dox and dox’ol in a cell. Thus, k_1_ is equivalent to the total dox and dox’ol in a cell for a given time.

We also calculated enzyme kinetics for dox using the enzyme kinetic Michaelis–Menten equation:

$$V = \frac{{V_{{{\text{max}}}} [Dox]}}{{K_{{\text{m}}} + [Dox]}}$$

V is velocity of conversion from dox to dox’ol; dox’ol formation at a given time. V_max_ is maximum rate of chemical conversion. [Dox] is the concentration of dox in a single cell or lysate. And, K_m_ is Michaelis–Menten constant.

Using a similar idea as in k_1_, we calculated rate constant of drug influx into cell nucleus. We simply measured the accumulation of dox (k_2_) and dox’ol (k’_2_) in the nucleus. The rate constant was calculated from the slope of total dox or total dox’ol accumulated in the nucleus. Thus, k_2_ and k’_2_ are equivalent to the respective total dox and total dox’ol in a nucleus for a given time.

## Supplementary Information

Supplementary Information
