# Supplementary material for: Label-free spectral imaging to study drug distribution and metabolism in single living cells
Source: Sci Rep. 2021 Feb 1;11:2703. doi: 10.1038/s41598-021-81817-0 (PMC7851119; doi:10.1038/s41598-021-81817-0)
Supplement: Supplementary file 1 — Supplementary Information [file 41598_2021_81817_MOESM1_ESM.pdf]

<u>Online Supplemental Figures</u>

Label-free spectral imaging to study drug distribution and metabolism in single living cells

Qamar A. Alshammari[1,2], Rajasekharreddy Pala[1,3], Nir Katzir[4], and Surya M. Nauli[1,3]

[1]Department of Biomedical & Pharmaceutical Sciences, Harry and Diane Rinker Health Science Campus, Chapman University, 9401 Jeronimo Road, Irvine, CA 92618-1908, USA.

[2]Department of Pharmacology and Toxicology, Faculty of Pharmacy, Northern Border University, KSA.

[3]Department of Medicine, University of California Irvine, Irvine, CA 92868, USA.

[4]Applied Spectral Imaging, 5315 Avenida Encinas, Suite 150, Carlsbad, CA 92008, USA.

Corresponding author:
Surya M. Nauli, Ph.D.
Chapman University
University of California Irvine
9401 Jeronimo Road.
Irvine, CA 92618-1908
Tel: 714-516-5480
Fax: 714-516-5481
Email: nauli@chapman.edu; snauli@uci.edu

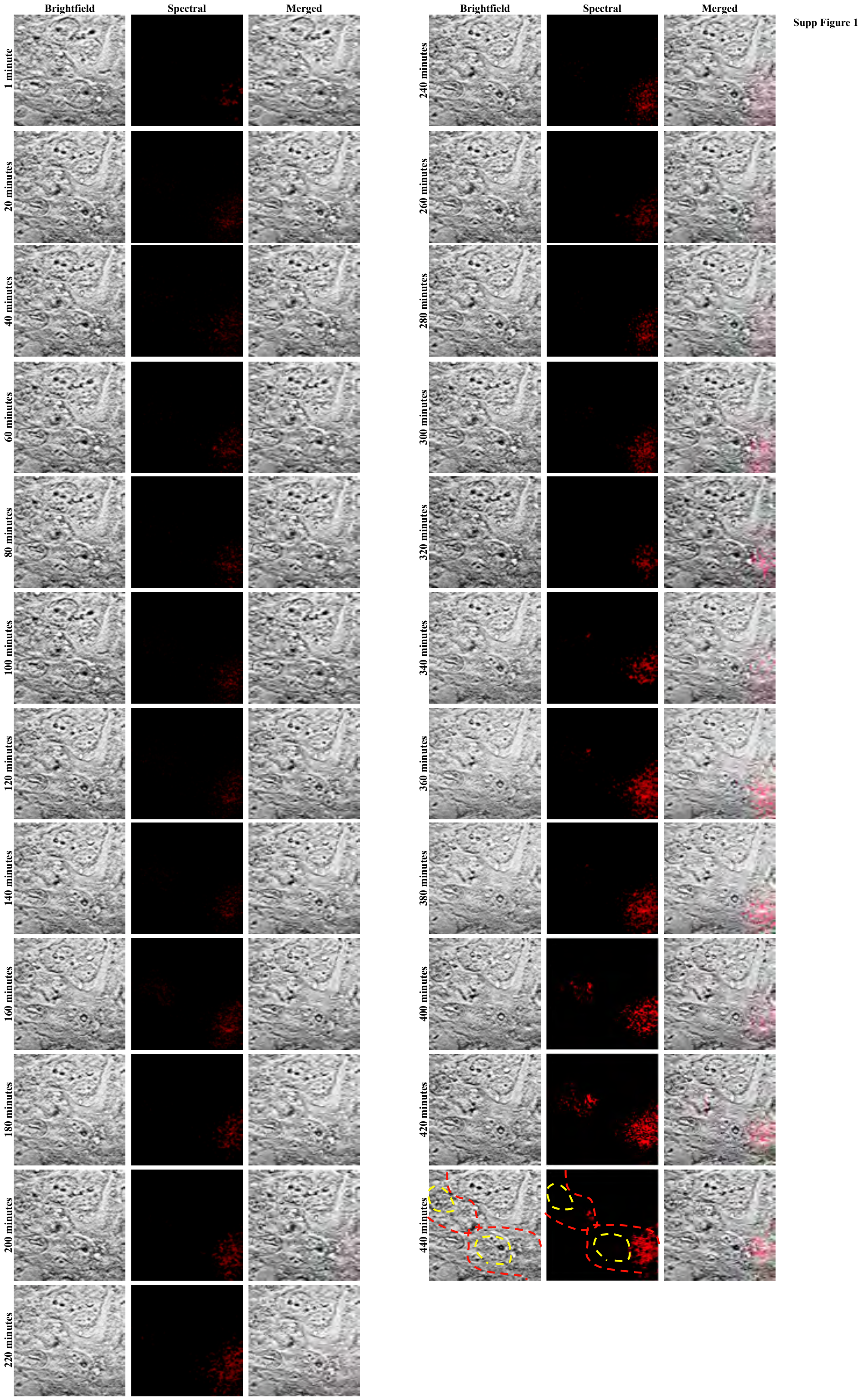

Supp Fig 1. Time-lapse imaging for the cells that treated with 0.01 nM rhodamine. Images were captured every 20 minutes for 440 minutes. Rhodamine accumulated in the cytoplasm more than in the nucleoplasm.

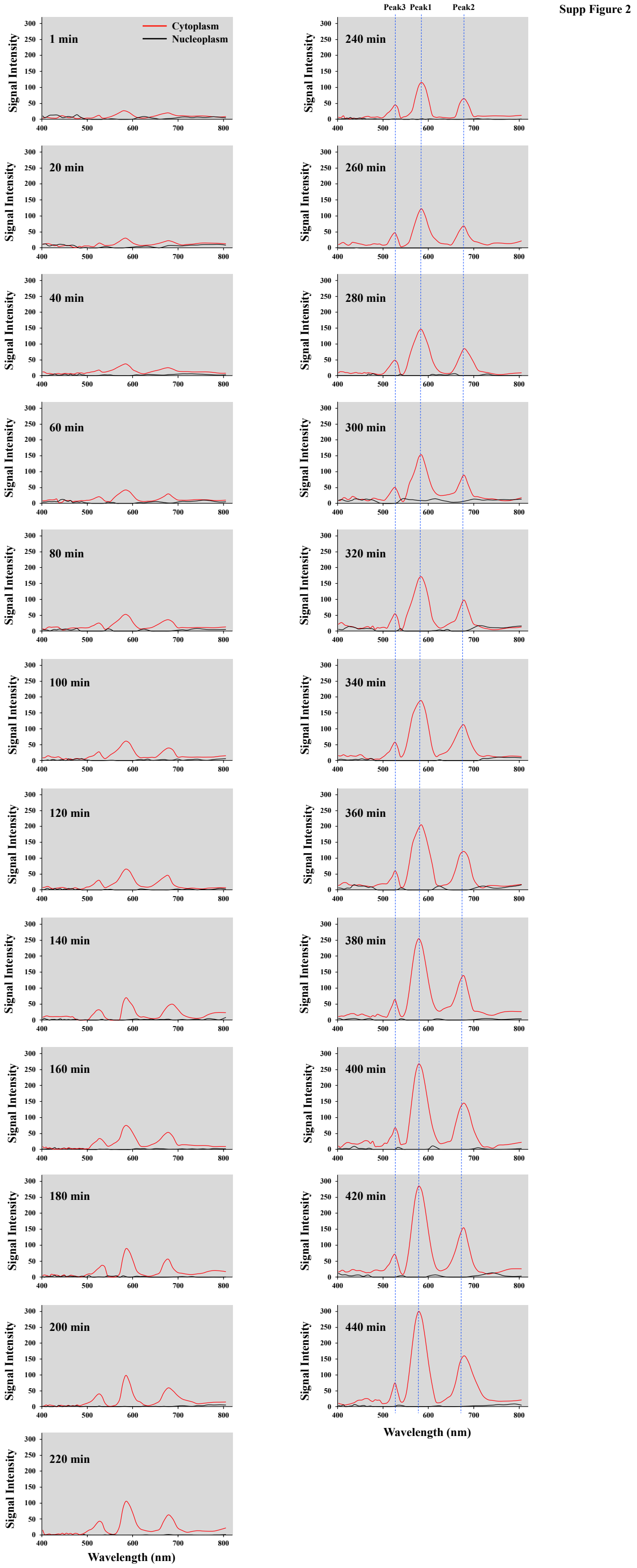

Supp Fig 2. Line graphs represent rhodamine wavelengths at different time points. The wavelength intensity increases over time. Red line represents the cytoplasm area, while black shows the background region, which is in the nucleoplasm.

Supp Figure 3

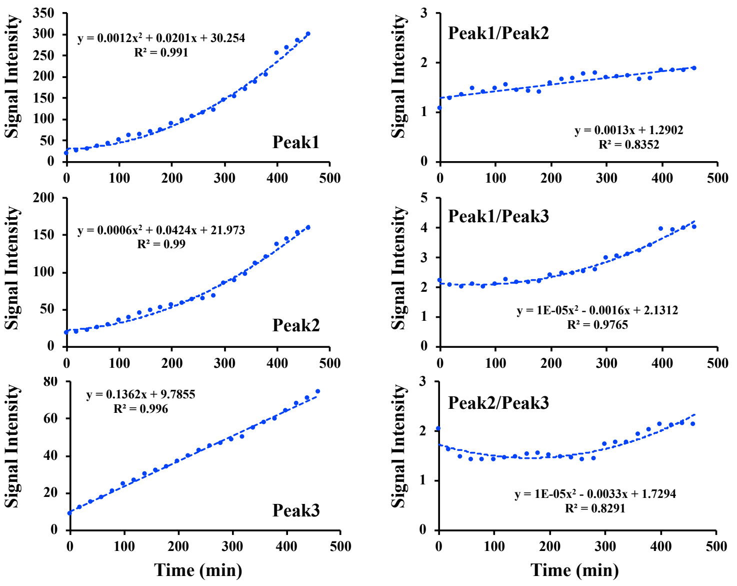

Supp Fig 3. The left panels show the analyses of time-lapse imaging data points for the cells treated with rhodamine for Peak1 (580 nm), Peak2 (679 nm) and Peak3 (515 nm). The right panels represent the ratios of different peaks.

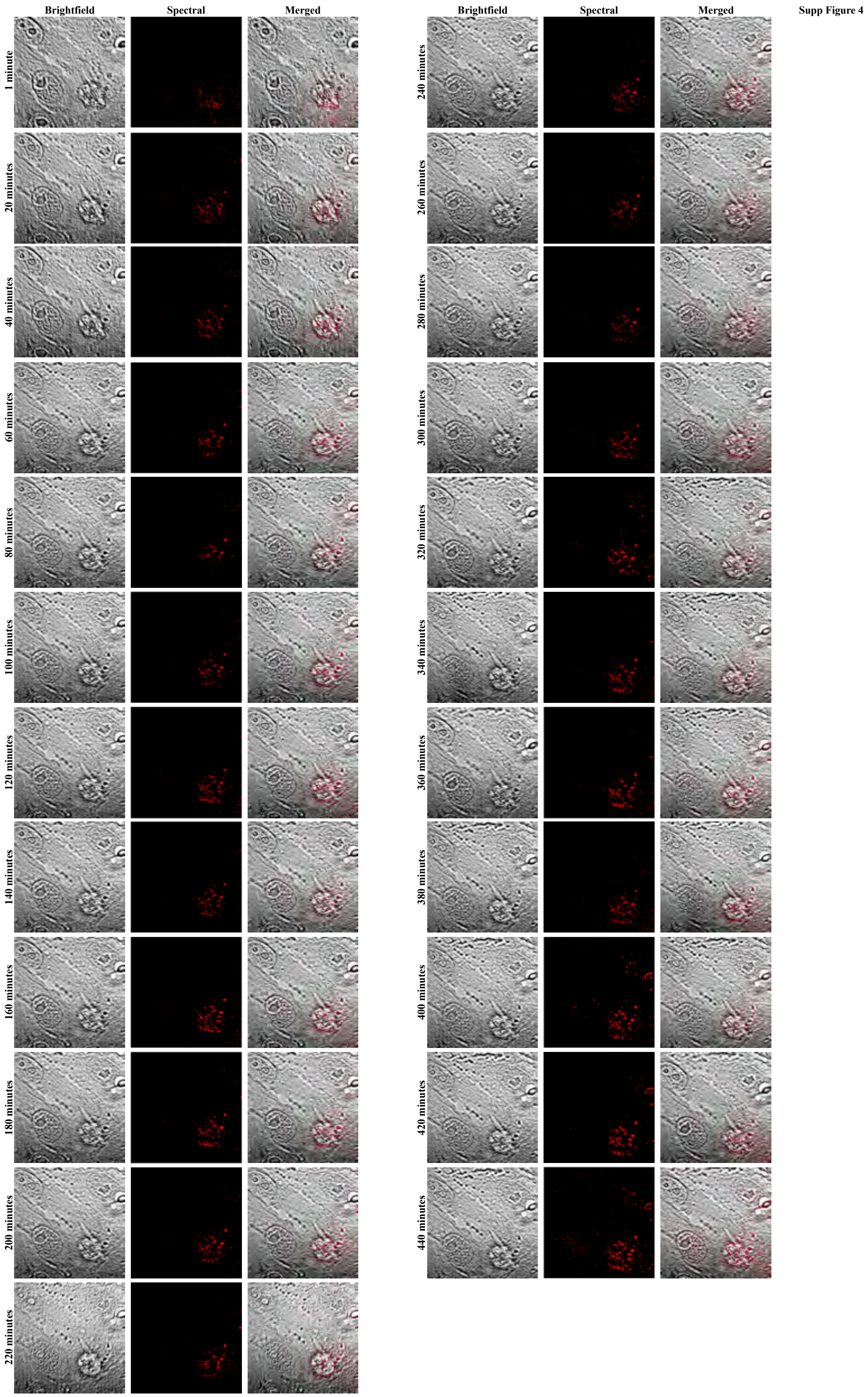

Supp Fig 4. Time-lapse imaging for the cells that treated with 1 nM dox. Images were captured every 20 minutes for 440 minutes. Dox accumulated in the nucleus area more than in the cytoplasm.

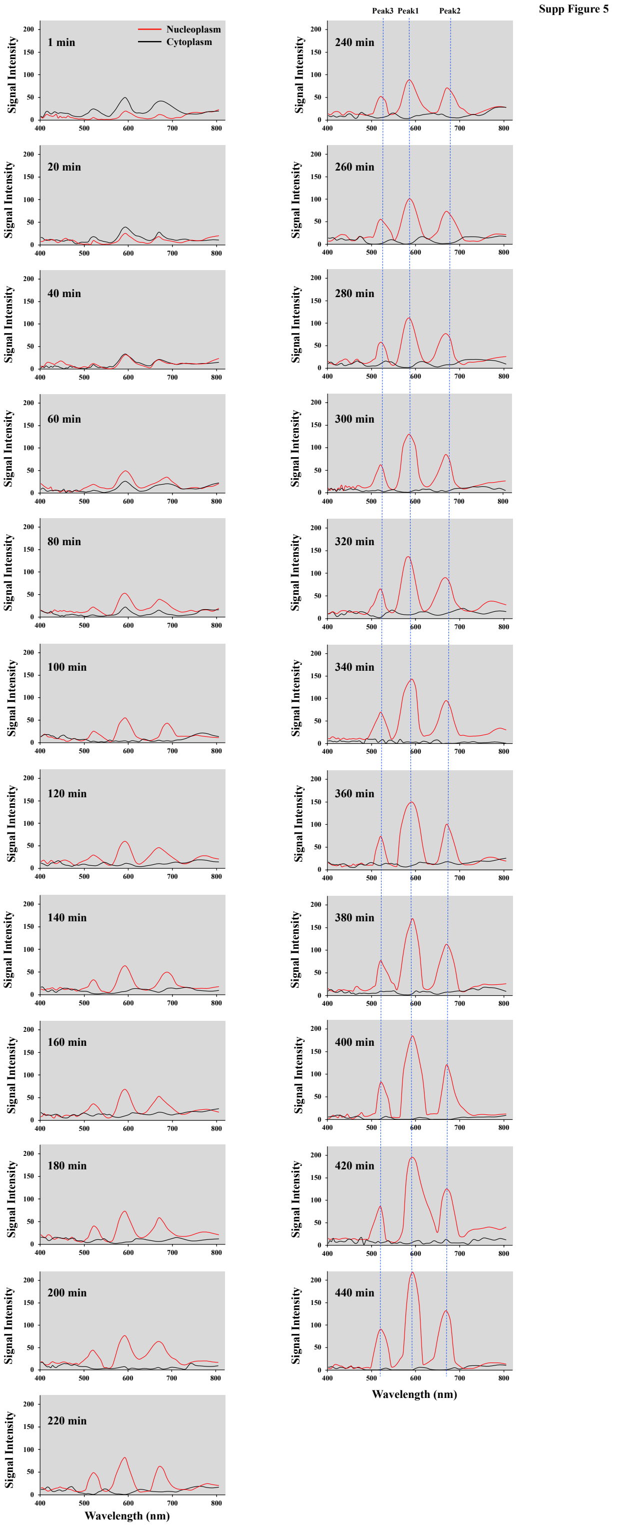

Supp Fig 5. Line graphs represent dox wavelengths at different time points. The wavelength intensity increases over time. The red line represents the nucleoplasm area, while black shows the background region, which is in the cytoplasm At the beginning of tracking, dox found in the cytoplasm area, but it then moved to the nucleoplasm area completely.

Supp Figure 6

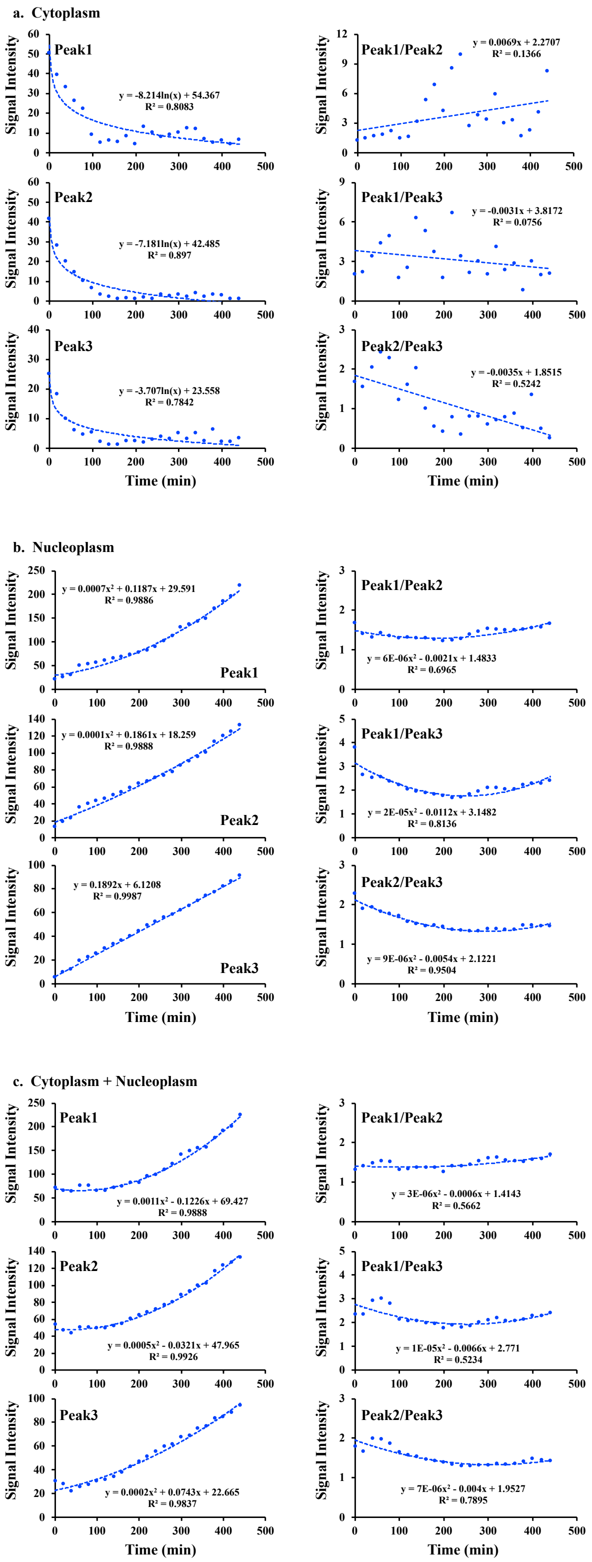

Supp Fig 6. Curve-fit analyses of time-lapse imaging for the cells treated with dox in cytoplasm (**a**), nucleoplasm (**b**) and entire cell (**c**). The left panels show the analyses of time-lapse imaging data points for the cells treated with rhodamine for Peak1 (592 nm), Peak2 (670 nm) and Peak3 (520 nm). The right panels represent the ratios of different peaks.

Supp Figure 7

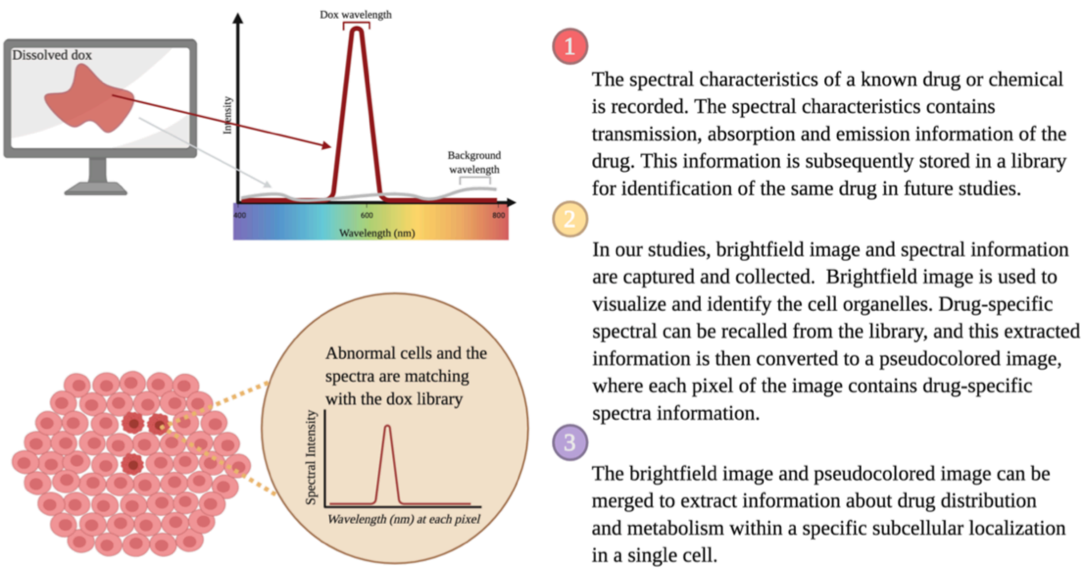

Supp Fig 7. Overview of the spectral imaging workflow. The first step is to make a spectral library for the chemical of interest. The second step is to take an image and collect its spectral information. The third step is to use the spectral library to identify spectral of the chemical of interest from the image.
